# Nourseothricin as a novel therapeutic agent against *Neisseria gonorrhoeae*: *in vitro* and *in vivo* evaluations using *Galleria mellonella*—a pilot study

**DOI:** 10.1128/spectrum.01275-25

**Published:** 2025-07-15

**Authors:** Izumo Kanesaka, Said Abdellati, Zina Gestels, Thibaut Vanbaelen, Irith De Baetselier, Tessa de Block, Sheeba Santhini Manoharan-Basil, Chris Kenyon

**Affiliations:** 1 Department of Clinical Sciences, Institute of Tropical Medicine Antwerp, Antwerp, Belgium; 2 Department of Infection Control and Prevention, Faculty of Nursing, Toho University, Tokyo, Japan; 3 University of Cape Town 37716, Cape town, South Africa; Michigan State University, East Lansing, Michigan, USA

**Keywords:** nourseothricin, streptothricin F, *Neisseria gonorrhoeae*, gonorrhea, *Galleria mellonella*

## Abstract

*Neisseria gonorrhoeae* has developed resistance to multiple antibiotics, making the search for novel antimicrobial agents essential. This pilot study evaluates the *in vitro* and *in vivo* efficacy of nourseothricin, a streptothricin family antibiotic mixture primarily composed of streptothricin F, against *N. gonorrhoeae*, a previously unexplored therapeutic option. The minimum inhibitory concentrations (MICs) of nourseothricin were determined using the agar dilution method on a panel of *N. gonorrhoeae* strains, including multidrug-resistant isolates. The *in vivo* efficacy was assessed using the *Galleria mellonella* infection model, where larvae were infected with *N. gonorrhoeae* and treated with varying concentrations of nourseothricin. Survival rates were compared to those of ceftriaxone-treated and untreated control groups. Nourseothricin inhibited the growth of all tested *N. gonorrhoeae* strains, with MIC values ranging from 16 to 32 µg/mL, independent of resistance to ceftriaxone, ciprofloxacin, or azithromycin. *In vivo*, treatment with nourseothricin significantly improved larval survival compared to the untreated control group (*P* < 0.01). The highest survival rate was observed with a 32 µg/mL dose, comparable to that of ceftriaxone-treated groups. Nourseothricin exhibited *in vitro* activity against *N. gonorrhoeae*, including multidrug-resistant strains. The *in vivo* results, obtained in a *Galleria mellonella* larval model, suggest that nourseothricin may warrant further investigation as a candidate compound for the treatment of gonorrhea, although its clinical potential remains to be evaluated in mammalian systems.

**IMPORTANCE:**

The emergence of multidrug-resistant *Neisseria gonorrhoeae* has made the development of new treatments a critical priority. This study is the first to evaluate the *in vivo* efficacy of nourseothricin against *N. gonorrhoeae*, demonstrating its activity even against resistant strains. In addition to its *in vitro* efficacy, treatment with nourseothricin improved survival in an *in vivo* infection model, suggesting its potential as a therapeutic option. Given the increasing limitations of existing antibiotics, these findings support further investigation into nourseothricin as a candidate for future gonorrhea treatment.

## INTRODUCTION

*Neisseria gonorrhoeae* is the causative agent of gonorrhea, a sexually transmitted infection that remains a significant global public health concern. Over the years, *N. gonorrhoeae* has acquired resistance to a variety of antibiotics, particularly penicillins, cephalosporins, and macrolides, complicating effective treatment strategies (1). The rapid evolution of *N. gonorrhoeae* and its ability to acquire resistance to multiple antibiotic classes make treatment increasingly challenging (2). Of particular concern, ceftriaxone-resistant gonococci have emerged globally, including recent reports from Europe and Asia (3). According to the 2021 United States Centers for Disease Control and Prevention (CDC) gonococcal (GC) treatment guidelines, ceftriaxone monotherapy is now recommended as preferred therapy, rather than combination ceftriaxone/azithromycin therapy due to increasing macrolide resistance (4). Ciprofloxacin is also not recommended as standard treatment because of widespread resistance. These guidelines are supported by the CDC’s Gonococcal Isolate Surveillance Project, which has documented persistently high levels of fluoroquinolone and macrolide resistance in the United States (5).

**Fig 1:**
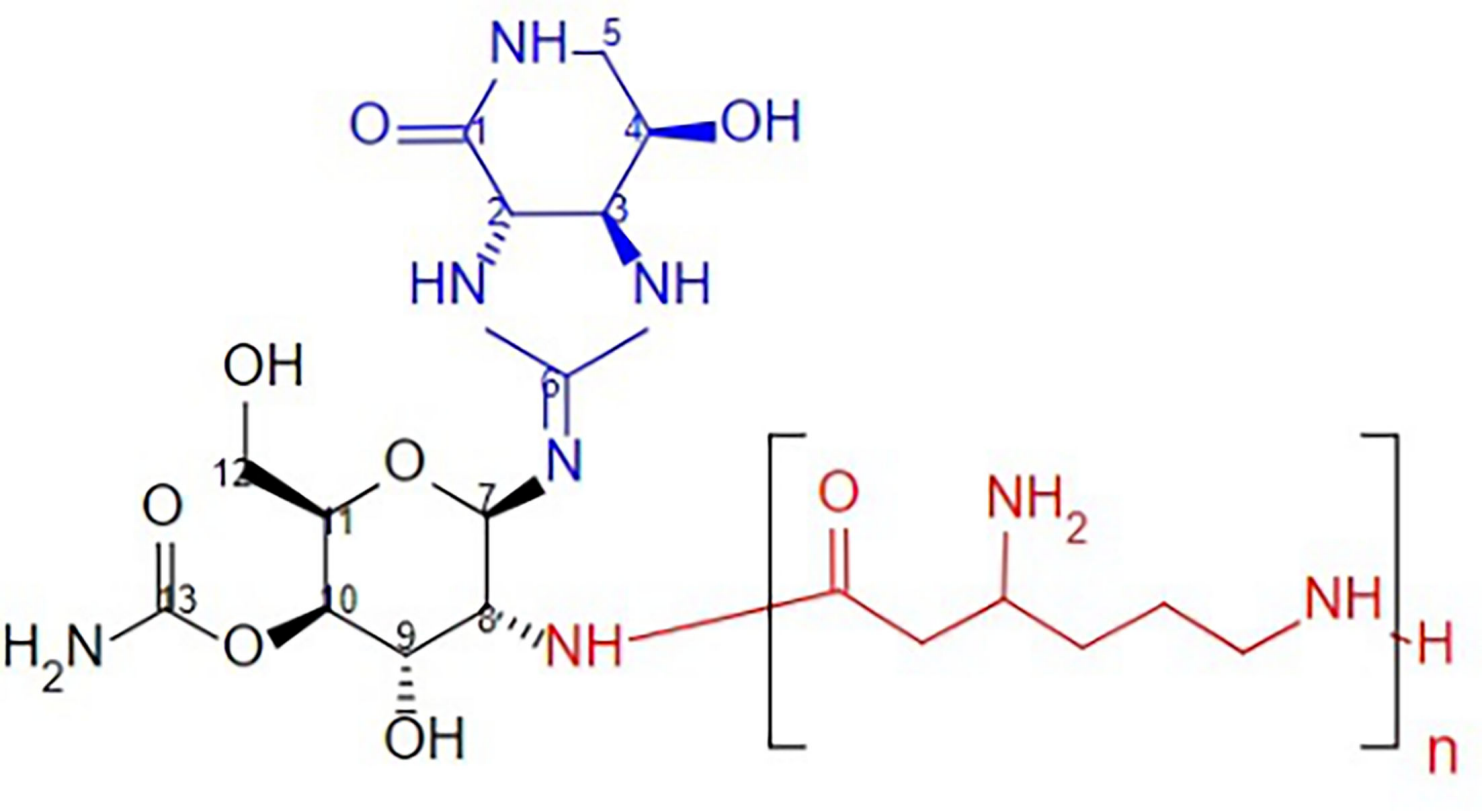
Structure of streptothricin family antibiotics. Streptothricins share a core architecture composed of a conserved streptolidine lactam ring (blue), a glucosamine sugar moiety (black), and a variable-length β-lysine tail (red). The number of β-lysine residues, ranging from one to seven, defines the specific streptothricin variant, such as streptothricin F (*n* = 1) to streptothricin X (*n* = 7). This figure was created using BKchem and adapted from structural data in Franck et al. (9).

Streptothricins represent a class of antibiotics derived from *Streptomyces* spp., exhibiting strong bactericidal activity, particularly against gram-negative bacteria, including multidrug-resistant (MDR) pathogens (6). Although both streptothricins and aminoglycosides target the 16S rRNA of the bacterial ribosome, streptothricins are structurally unrelated and interact with different binding sites, resulting in distinct mechanisms of action. This distinction is particularly relevant because aminoglycosides such as gentamicin have been explored as alternative treatments for drug-resistant *Neisseria gonorrhoeae* (7). Thus, understanding these mechanistic differences supports the rationale for investigating streptothricin F (STC-F) as a novel therapeutic option, especially in the context of rising antimicrobial resistance. A recent study by Morgan et al. (8) using cryogenic electron microscopy revealed that streptothricins F and D primarily bind to helix 34 of the 16S rRNA of the 30S ribosomal subunit. This interaction stabilizes non-cognate tRNAs at the A site, leading to lethal translational errors and contributing to their bactericidal activity against highly drug-resistant gram-negative bacteria (8). Streptothricins are a class of antimicrobial compounds characterized by three key structural components: (i) a streptolidine lactam ring (Fig. 1, blue component); (ii) a glucosamine sugar (Fig. 1, black component); and (iii) a β-lysine homopolymer, which varies in length from a single β-lysine residue to up to seven residues (Fig. 1, red component). These structural features were elucidated through chemical degradation studies conducted in 1952, 1956, and 1961 (9), confirming that different streptothricin analogs (formerly referred to as yazumycins and racemomycins) primarily differ in the number of β-lysine residues attached to the glucosamine sugar. Streptothricins exert their antibacterial activity by inhibiting protein synthesis, primarily through interactions with the ribosome. The length of the β-lysine homopolymer has been shown to influence antibacterial potency, with longer chains generally associated with increased activity. Notably, streptothricins display potent activity against certain gram-negative bacteria, including MDR pathogens, highlighting their potential as a novel therapeutic option in the face of rising antimicrobial resistance.

Streptothricins are a structurally diverse group, with different antibacterial potency and toxicity profiles. Among them, streptothricin F has gained attention due to its improved efficacy and reduced toxicity compared to earlier-generation compounds such as streptothricins D and E (10). While the early derivatives showed nephrotoxicity in mammalian systems, streptothricin F has been reported to exhibit a more favorable therapeutic index, making it a promising candidate for further evaluation in clinical applications (8). Recent studies have established that the murine LD50 of streptothricin F is 300 mg/kg, which is higher than those for streptothricin D (STC-D) (10 mg/kg) and gentamicin (52 mg/kg) (11, 12). Morgan et al. have demonstrated that streptothricin F exhibits strong activity against carbapenem-resistant *Escherichia coli* and *Klebsiella pneumoniae* (8). However, its effectiveness against *N. gonorrhoeae* has not been systematically investigated. Considering the urgent need for alternative antimicrobial agents, streptothricin F’s broad-spectrum activity and lower toxicity profile make it an attractive candidate for combating drug-resistant gonorrhea. In this study, we therefore systematically examined the *in vitro* and *in vivo* efficacy of streptothricin F against *N. gonorrhoeae*, providing insights into its therapeutic potential and feasibility as a new treatment option.

## MATERIALS AND METHODS

### *In vivo* infection model using *Galleria mellonella*

The *Galleria mellonella* (greater wax moth) larval model has been increasingly used in antimicrobial research due to its low cost, ethical advantages, and conserved innate immune responses comparable to those of mammals (13). However, it is important to note that *N. gonorrhoeae* is a strict human pathogen with limited ability to establish infection in non-human hosts. Previous studies have shown that *N. gonorrhoeae* does not replicate within *G. mellonella* and that CFU counts decline over time. Furthermore, the larvae lack human-specific receptors critical for gonococcal pathogenesis. Studies that have applied this model to *N. gonorrhoeae* (including references 13 and 14) also explicitly acknowledge these limitations. We have accordingly interpreted our results in light of these model-specific constraints. Despite these limitations, injection of *N. gonorrhoeae* into the larval hemocoel results in progressive melanization and increased mortality over 72–96 h, offering a reproducible platform for preliminary therapeutic evaluation. Therefore, this model was used in the present study (14).

### *Neisseria gonorrhoeae* isolates

To assess the effects of nourseothricin on *N. gonorrhoeae*, a panel of 16 clinical isolates and four reference strains (American Type Culture Collection [ATCC] 49226 and World Health Organization [WHO] X, Y, and Z) was chosen. Further detailed information on the bacterial strains used in this study is provided in Table 1. As noted above, ceftriaxone resistance is a high-priority concern. As a result, these isolates were selected primarily because of their high ceftriaxone minimum inhibitory concentrations (MICs). More specifically, the three WHO strains have the highest ceftriaxone MICs in the WHO panel, and 12 of the 16 clinical isolates were selected based on the fact that they had the highest ceftriaxone MICs in our collection of clinical isolates (all ≥0.19 µg/mL). In addition, we selected four clinical isolates and ATCC 49226 that had low ceftriaxone MICs (all ≤0.008 µg/mL). These isolates had a range of ciprofloxacin (from <0.002 to >32 µg/mL) and azithromycin MICs (0.032–1.0 µg/mL, Table 1).

**TABLE 1:** Nourseothricin MICs (µg/mL)^*a*^^*,*^^*b*^

Strain ID	MIC (µg/mL)			
	Ceftriaxone	Ciprofloxacin	Azithromycin	Nourseothricin
15441	0.25	16.0	0.25	32
17155	0.25	4.0	0.5	32
17360	0.25	16.0	0.5	32
18227	0.19	>32.0	1.0	16
18243	0.19	16.0	0.25	16
18293	0.19	8.0	0.25	32
19598	0.5	>32.0	0.38	16
20090	0.19	>32.0	0.25	32
20442	0.19	>32.0	0.75	32
21140	0.25	>32.0	0.75	32
21272	0.19	>32.0	1.0	32
23509	0.19	6.0	0.25	32
24758	<0.002	<0.002	0.032	32
24772	0.004	3.0	1.5	32
24773	0.003	0.002	1.0	32
24774	<0.002	0.002	0.5	32
ATCC 49226	0.008	0.002	0.25	16
WHO X	2.0	>32.0	0.5	32
WHO Y	1.0	>32.0	1.0	32
WHO Z	0.5	>32.0	1.0	16

^*a*^ EUCAST breakpoints (2024) for *N. gonorrhoeae* are as follows: ceftriaxone, S ≤ 0.125 µg/mL; ciprofloxacin, S ≤ 0.03 µg/mL; and azithromycin, S ≤ 0.5 µg/mL. No established breakpoints are currently available for nourseothricin.

^*b*^ MIC, minimum inhibitory concentration.

### Antimicrobial susceptibility testing

The agar dilution test was performed using GC agar base (BD Difco, Belgium) as the medium, supplemented with 1% IsoVitaleX (BD, Belgium), in accordance with the Clinical and Laboratory Standards Institute agar dilution method (15). The *N. gonorrhoeae* strains were grown on Columbia blood agar with 5% sheep blood and incubated at 36°C with 5% CO_2_ overnight. The culture of each strain was then used to make a 0.5 McFarland (McF) suspension in a tube with 2 mL of Mueller-Hinton broth (BD Difco). A dilution of the suspension of approximately 10^4^ CFU per spot was inoculated within 15 min of preparation onto the agar surface with a multipoint inoculator. The plates were incubated at 37°C in 5% CO_2_ for 20–24 h. The MICs were interpreted by reading growth inhibition.

### Preparation of live microbial inocula for infection

Clinical isolate *N. gonorrhoeae* id: 19598 was selected to infect the larvae. This strain was a multidrug-resistant strain with ceftriaxone, ciprofloxacin, and nourseothricin MICs of 0.5, >32, and 16 µg/mL, respectively. The test strain was cultured from frozen stocks onto GC-Lect medium (BD BBL) for ≤16 h at 37°C with 5% (vol/vol) CO_2_. Single colonies were selected from these cultures and spread onto fresh agar plates, which were incubated at 37°C with 5% (vol/vol) CO_2_ overnight. Preparation of the test strains was carried out according to established methods (16). The *N. gonorrhoeae* isolate was then inoculated into the hemocoel of the larva (30 µL of phosphate-buffered saline [PBS] containing 6 McF). This dose of *N. gonorrhoeae* was determined based on a previous study we conducted, which found that this dose of *N. gonorrhoeae* (id: 19598) resulted in an optimal mortality rate for assessing the therapeutic efficacy of novel antigonococcal agents.

### Injection of microbial inocula of *Galleria mellonella* larvae

Last larval stage *G. mellonella* (Terramania, Arnhem, Netherlands) were used for the experiments. Only macroscopically healthy, non-discolored larvae weighing 300–450 mg were selected. The larvae were placed into individual sterile Petri dishes in groups of 10 per Petri dish. The larvae were kept in an incubator at 37°C with a 5% (vol/vol) CO_2_ atmosphere for the length of the experiments. The larvae were injected in the last right pro-leg with 30 µL of bacterial suspension. Each group consisted of 10 larvae per replicate, and the experiments were independently repeated three times, resulting in a total of 30 larvae per group.

### Concentration of nourseothricin and ceftriaxone injected

**Fig 2:**
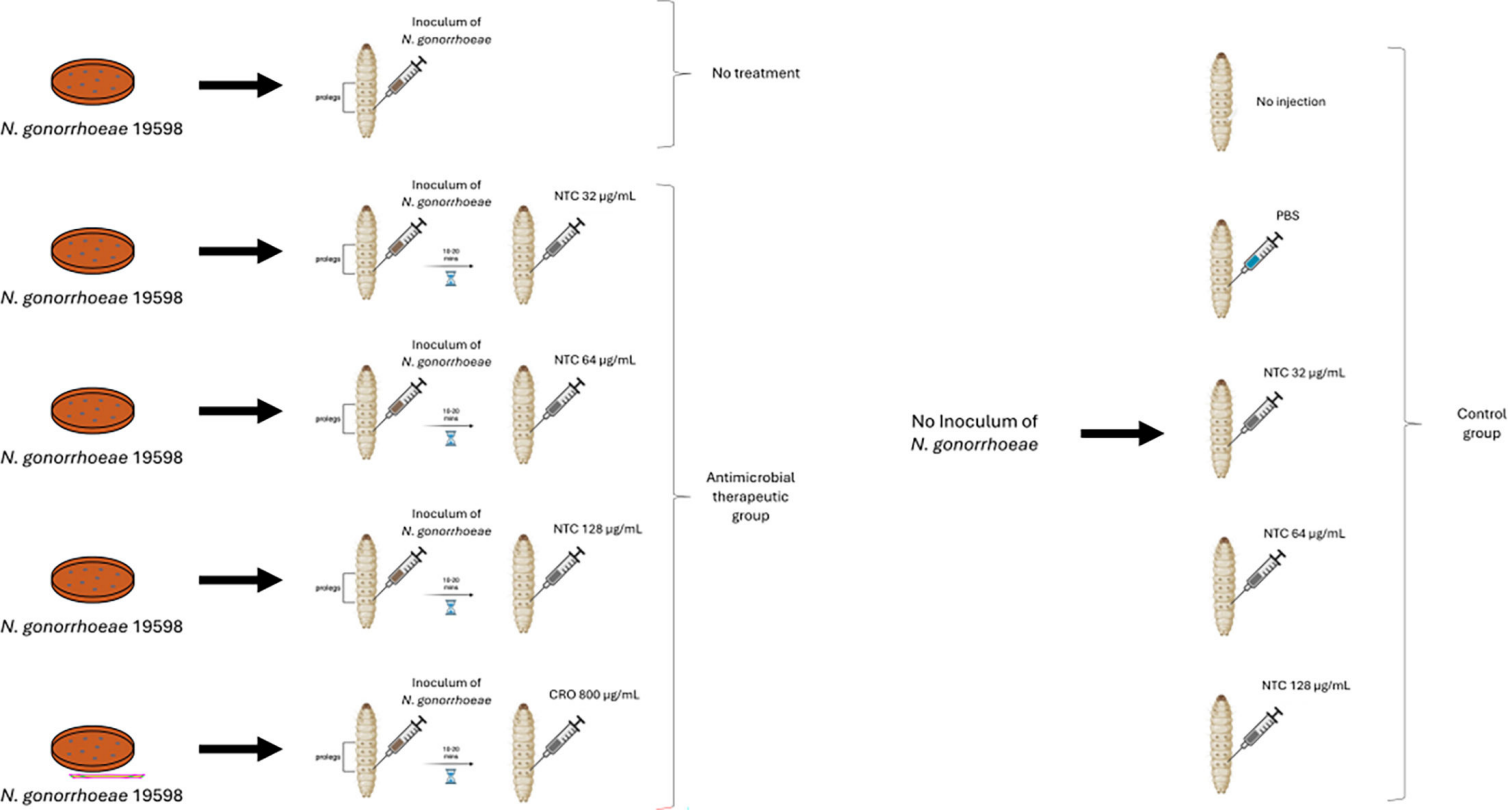
Experimental design of the *Galleria mellonella* infection model with *N. gonorrhoeae*. Larvae were infected with a multidrug-resistant clinical isolate of *N. gonorrhoeae* (19598), followed by treatment with nourseothricin or ceftriaxone. Control groups included larvae that received no injection or PBS only. Toxicity groups received antimicrobial treatment without bacterial inoculation. Thirty larvae were used per condition.

Ten to twenty minutes after injection of the bacterial suspension, the larvae were injected with various doses of nourseothricin (MedChemExpress, Sweden). It was used in all experiments. Nourseothricin is a streptothricin family antibiotic mixture that typically contains approximately 65% STC-F, 30% STC-D, and 5% streptothricin E (STC-E) (8). We refer to this compound as nourseothricin throughout the article, acknowledging that STC-F is the major active component. While STC-F is considered the major active antimicrobial component, STC-D and STC-E may contribute disproportionately to toxicity in mammalian systems (8). It was dissolved in distilled water for all experiments. The larvae were injected with the various antimicrobials and PBS using 0.3 mL U-100 insulin syringes (BD Micro-Fine). One syringe and needle were used for 10 larvae in each Petri dish. We tested three concentrations of nourseothricin, 32, 64, and 128 µg/mL. These doses were based on the nourseothricin MIC of 16–32 µg/mL established on agar dilution testing. An *N. gonorrhoeae* control group received an injection of ceftriaxone (20 mg/kg). This dose of ceftriaxone was based on the results of a previous study, which found this dose of ceftriaxone to be efficacious against *N. gonorrhoeae* WHO P in *G. mellonella* (17). To achieve this dose, larvae with an average body weight of approximately 0.4 g were injected with 10 µL of ceftriaxone at a concentration of 800 µg/mL. Assuming a hemolymph volume proportional to body weight, this corresponds to an estimated initial hemolymph concentration of ~80 µg/mL, which considerably exceeds the ceftriaxone MIC for *N. gonorrhoeae* id: 19598 (MIC 0.5 µg/mL). This dose and injection volume are consistent with previous studies using *Galleria mellonella* as an *in vivo* infection model (18, 19). Two types of negative control groups were included to distinguish the effects of injection trauma from those of bacterial infection or drug treatment: (i) a no-injection group and (ii) a group injected with 30 µL of PBS. Three toxicity groups were evaluated that received only 10 µL of each drug concentration (32, 64, and 128 µg/mL) without bacterial inoculation. The design of the *Galleria mellonella* infection model, including antimicrobial therapeutic and control groups, is illustrated in Fig. 2.

The larvae were assessed at 24, 48, 72, and 96 h post-inoculation for mortality (defined as a lack of movement in response to tactile stimulus (14)). When each experiment was completed, both the surviving and dead *G. mellonella* were kept at −80°C overnight to sedate and kill them. They were then autoclaved at 121°C for 15 min and discarded.

### Data analysis

Statistical analyses and data visualization were conducted using GraphPad Prism (version 10.4). Depending on the Gaussian distribution of the data, assessed using the Shapiro-Wilk test, comparisons between groups were performed using either the Mann-Whitney test or analysis of variance. The survival curves were analyzed using both the log-rank (Mantel-Cox) test and the Gehan-Breslow-Wilcoxon test to assess statistical significance. A *P* value of <0.01 was considered statistically significant.

## RESULTS

### Minimum inhibitory concentrations

The MICs for the *N. gonorrhoeae* isolates are provided in Table 1. The nourseothricin MIC_50_, MIC_90_, and MIC_range_ were 32, 32, and 16–32 µg/mL, respectively. Specifically, five strains exhibited an MIC of 16 µg/mL, while all other strains showed an MIC of 32 µg/mL. The MICs of ceftriaxone varied among the tested strains, with the lowest values observed in 24758, 24772, 24773, and 24774 (≤0.004 µg/mL) and the highest in 19598 (0.5 µg/mL). Ciprofloxacin resistance was notably high in 18227, 19598, 20090, 20442, 21140, and 21272 (>32 µg/mL), whereas azithromycin MICs ranged from 0.032 µg/mL in 24785 to 1 µg/mL or higher in 18227, 21272, 24772, and 24773. There was no association between MICs of nourseothricin and any of the other three antimicrobials assessed (ceftriaxone, rho −0.113; ciprofloxacin, −0.169; and azithromycin, 0.031).

### Mortality

**Fig 3:**
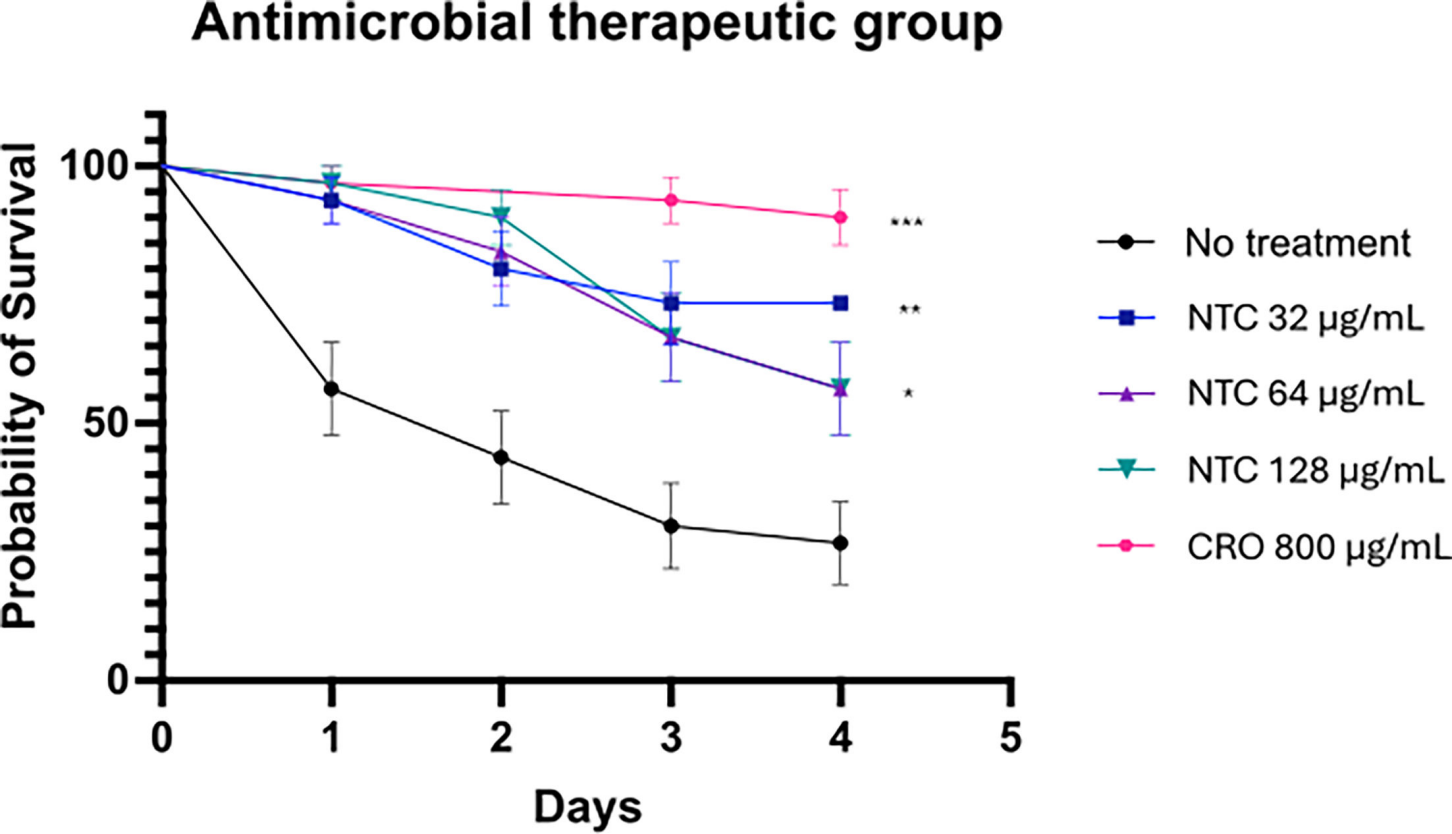
Survival curve of the antimicrobial therapeutic group. Survival curve of *Galleria mellonella* infected with *N. gonorrhoeae* (6 McF) and treated with varying doses of nourseothricin, ceftriaxone, or PBS. ***P < 0.0001, **P < 0.001, *P < 0.01(comparisons with “no treatment“ group). Symbols in each panel represent the mean survival from three independent experiments, and the error bars represent the standard deviation of the mean. CRO, ceftriaxone; NTC, nourseothricin.

The ceftriaxone-treated group as well as the 32, 64, and 128 µg/mL nourseothricin-treated groups had a statistically significant increase in survival compared to the no-treatment group (all *P* ≤ 0.01; Fig. 3).

### Toxicity

**Fig 4:**
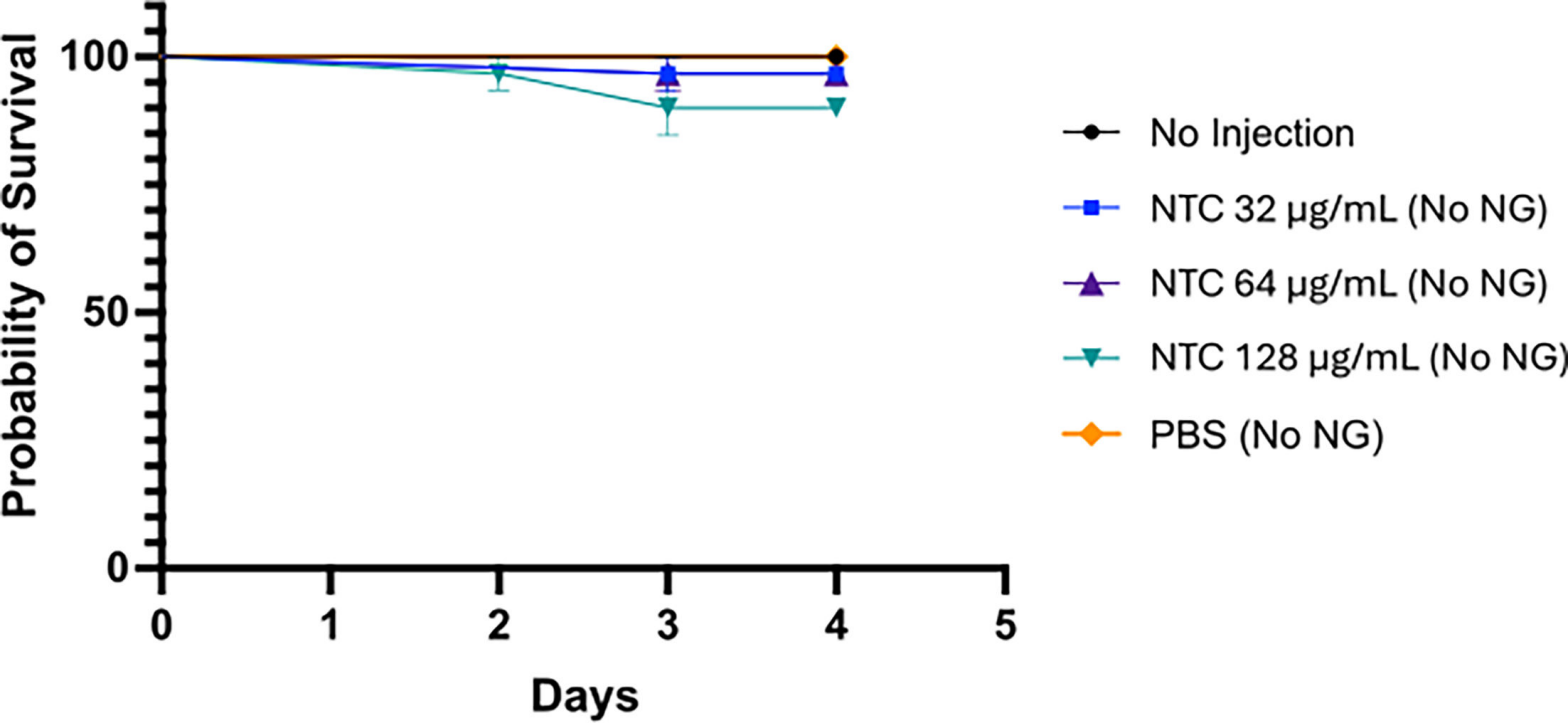
Toxicity of nourseothricin. To assess nourseothricin toxicity, the impact of different concentrations of nourseothricin (32, 64, or 128 µg/mL) on larval survival was compared with that of larvae that received no injection and larvae injected with 30 µL of PBS. Survival proportions for these groups were recorded over 4 days, and the probability of survival was analyzed accordingly. NTC, nourseothricin; PBS, phosphate-buffered saline.

There was no difference in mortality between the larvae injected with nourseothricin and those injected with PBS (Fig. 4). The probability of survival was ≥80% up to day 4 in all conditions assessed (Fig. 4). Larval health changes were monitored using the *G. mellonella* Health Index Scoring System based on Loh et al. (20). No melanization was observed in larvae following the receipt of nourseothricin.

## DISCUSSION

In this study, we evaluated the antimicrobial efficacy of nourseothricin against clinical and reference isolates of *Neisseria gonorrhoeae*. The MIC values of nourseothricin indicate a relatively narrow MIC distribution (from 16 to 32 µg/mL) for nourseothricin among the tested strains and were not associated with the MICs of ceftriaxone, ciprofloxacin, or azithromycin. Additionally, *in vivo* experiments using *Galleria mellonella* revealed that treatment with nourseothricin at 32 µg/mL resulted in the highest survival rate, followed by the ceftriaxone (20 mg/kg) group. Treatment with nourseothricin at 64 and 128 µg/mL also improved survival compared to the untreated control group, but to a lesser extent. Interestingly, the highest survival rate was observed at the lowest nourseothricin dose tested (32 µg/mL), with no apparent dose-response trend at higher concentrations. This may suggest early saturation of the therapeutic effect, limitations of the larval model, or possible toxicity at higher doses that is undetectable in this system. These findings suggest that future studies could assess 32 µg/mL nourseothricin in mammalian models of gonococcal infection.

Previous studies have demonstrated the antimicrobial activity of streptothricins, especially against drug-resistant bacteria. Morgan et al. reported that nourseothricin effectively inhibits highly drug-resistant gram-negative bacteria, including a pan drug-resistant *Klebsiella pneumoniae* strain, by inhibiting the 30S ribosomal subunit (8). These findings align with our results and support the notion that nourseothricin retains activity against strains resistant to conventional antibiotics. This is particularly relevant in light of the increasing prevalence of gonococcal antibiotic resistance. The rise in resistance to ceftriaxone and azithromycin has made the development of novel antibiotics a critical public health priority (21).

A limitation of this study is the use of the *Galleria mellonella* model for *in vivo* experiments. While this model is valuable for initial antimicrobial testing, it does not fully replicate the complexity of human infections (13). *Galleria mellonella* is of only limited utility in toxicity assessment. While this invertebrate model has proven useful for initial screening of general antimicrobial efficacy and acute toxicity, it lacks mammalian organ systems such as kidneys, which are the primary target organs for streptothricin-associated toxicity (22). As such, the absence of toxicity signals in this model cannot be interpreted as evidence of renal safety. Thus, further research, including studies with mammalian infection models and clinical trials in humans, is needed to validate the therapeutic potential of nourseothricin.

Additionally, we only evaluated the effect of nourseothricin on a limited number of clinical and reference isolates of *N. gonorrhoeae*. Although resistance development was not experimentally evaluated in this study, several aspects of streptothricin resistance are reviewed here to provide context and guide future investigation. Importantly, we did not assess the potential for the emergence of gonococcal resistance to nourseothricin. Previous studies in *Escherichia coli* have found that 16S rRNA resistance-associated mutations only emerged in strains that only had a single copy of the 16S rRNA operon but not in wild-type *E. coli* that have seven 16S rRNA operons (8). The C1054A, A1196C, and A1196G mutations in 16S rRNA were responsible. The fact that *N. gonorrhoeae* had four copies of the 16S rRNA operon may reduce the probability of this type of resistance emerging in *N. gonorrhoeae* (23). Other studies have also reported the emergence of streptothricin-resistant bacterial strains (24). Resistance to streptothricin has been primarily associated with enzymatic modifications, such as acetylation of the β-lysine moiety and hydrolysis of the streptolidine lactam ring, mediated by specific aminoglycoside-modifying enzymes and streptothricin-specific hydrolases (9, 25). The presence of these resistant strains raises concerns about the long-term clinical efficacy of nourseothricin.

Historically, streptothricins were widely used as growth promoters in pig farming in former East Germany from 1981 to 1988 (24). Soon after starting this practice, streptothricin-resistant *E. coli* were isolated from pigs, agricultural sewage, and even fecal samples from agricultural workers at those farms. The streptothricin resistance was shown to be caused by plasmids encoding multidrug-resistant transposon cassettes with genes for a streptothricin acetyltransferase and an aminoglycoside adenyltransferase (26, 27). This example provides an important reminder of the potential risk of rapid dissemination of streptothricin resistance if it is reintroduced for clinical use. It is unknown if the acetyl- and adenyl-transferase-containing transposons could induce streptothricin resistance in *N. gonorrhoeae*.

The MIC_90_ in our study (32 µg/mL) was higher than that found for carbapenem-resistant *Enterobacterales* (4 µM, approximately 2 µg/mL). Studies in mice have found that the lowest nourseothricin dose at which mild nephrotoxicity was detected was 100 mg/kg (8). While animal studies suggest lower toxicity of nourseothricin compared to other antibiotics, data on human toxicity remain limited.

The replicable delayed toxicity observed in streptothricin and streptothricin-like antibiotics in mammalian test organisms led to delayed toxicity being recognized as a defining characteristic of this antimicrobial class. This phenomenon was further characterized by Inamori et al., who conducted comprehensive toxicity studies in both mice and rats (11). Their findings demonstrated that, following intravenous administration in mice, nourseothricin predominantly accumulates in renal tissues, with minimal active antibiotic recovered from urine samples. Histological analyses of kidney tissues from treated animals confirmed streptothricin-induced nephrotoxicity. Prior to the identification of this delayed toxicity, the pharmaceutical company Merck initiated a clinical trial in humans in which all four participants experienced acute urinary retention (28). Although these historical and preclinical data provide important information about streptothricin toxicity, there are currently no clinical data available regarding the safety or adverse effects of nourseothricin in humans. The severe nephrotoxicity, in conjunction with the emergence of less toxic broad-spectrum antibiotics, ultimately led to the abandonment of streptothricins for clinical development.

Subsequent efforts to mitigate the toxicity of streptothricins have yielded partial success. Enzymatic hydrolysis of streptothricin D via a streptothricin hydrolase resulted in a 32-fold reduction in antimicrobial activity against *Escherichia coli* while concurrently producing a 128- to 256-fold reduction in cytotoxicity in eukaryotic cells, suggesting a potential medicinal chemistry approach to reducing toxicity (29). Furthermore, toxicity studies in mice have demonstrated a direct correlation between the length of the β-lysine chain and toxicity, with nourseothricin (containing a single β-lysine residue) exhibiting significantly lower toxicity than streptothricin D (harboring three β-lysine residues) as reflected in their respective LD50 values of 300 and <10 mg/kg. This differential toxicity has been hypothesized to be linked to variations in cellular internalization.

Future studies are required to assess not only the toxicity but also the penetration of streptothricin to key target tissues colonized by *N. gonorrhoeae*, such as the pharynx, urethra, cervix, and anorectum. In particular, the penetration into the pharynx will be important to evaluate since other similarly hydrophilic antimicrobials, such as aminoglycosides, have been noted to have poor penetration at this site (30). This has translated into poorer efficacy at clearing pharyngeal infections (31). Future studies could evaluate for synergy or antagonism with other therapies for *N. gonorrhoeae*. Combination therapy with standard treatments such as ceftriaxone may be of use in settings such as Vietnam, where recent studies have found a very high prevalence of ceftriaxone resistance (32).

### Conclusion

Nourseothricin demonstrated antimicrobial activity against drug-resistant *N. gonorrhoeae* and improved survival in a *Galleria mellonella* larval infection model. While these findings are encouraging, the limitations of this invertebrate model must be recognized. Further studies using mammalian infection models are needed to validate its efficacy and evaluate safety before any consideration of clinical application.
